# Structural basis for the specificity of renin-mediated angiotensinogen cleavage

**DOI:** 10.1074/jbc.RA118.006608

**Published:** 2018-12-18

**Authors:** Yahui Yan, Aiwu Zhou, Robin W. Carrell, Randy J. Read

**Affiliations:** From the ‡ Department of Haematology, University of Cambridge, Cambridge Institute for Medical Research, Wellcome Trust/MRC Building, Hills Road, Cambridge CB2 0XY, United Kingdom and; the § Hongqiao International Institute of Medicine, Shanghai Tongren Hospital/Faculty of Basic Medicine, Key Laboratory of Cell Differentiation and Apoptosis of the Chinese Ministry of Education, Shanghai Jiao Tong University School of Medicine, Shanghai 200025, China

**Keywords:** renin angiotensin system, serpin, crystal structure, conformational change, site-directed mutagenesis, kinetics, proteolysis, aspartic protease, hypertension, angiotensinogen

## Abstract

The renin–angiotensin cascade is a hormone system that regulates blood pressure and fluid balance. Renin-mediated cleavage of the angiotensin I peptide from the N terminus of angiotensinogen (AGT) is the rate-limiting step of this cascade; however, the detailed molecular mechanism underlying this step is unclear. Here, we solved the crystal structures of glycosylated human AGT (2.30 Å resolution), its encounter complex with renin (2.55 Å), AGT cleaved in its reactive center loop (RCL; 2.97 Å), and spent AGT from which the N-terminal angiotensin peptide was removed (2.63 Å). These structures revealed that AGT undergoes profound conformational changes and binds renin through a tail-into-mouth allosteric mechanism that inserts the N terminus into a pocket equivalent to a hormone-binding site on other serpins. These changes fully extended the N-terminal tail, with the scissile bond for angiotensin release docked in renin's active site. Insertion of the N terminus into this pocket accompanied a complete unwinding of helix H of AGT, which, in turn, formed key interactions with renin in the complementary binding interface. Mutagenesis and kinetic analyses confirmed that renin-mediated production of angiotensin I is controlled by interactions of amino acid residues and glycan components outside renin's active-site cleft. Our findings indicate that AGT adapts unique serpin features for hormone delivery and binds renin through concerted movements in the N-terminal tail and in its main body to modulate angiotensin release. These insights provide a structural basis for the development of agents that attenuate angiotensin release by targeting AGT's hormone binding pocket.

## Introduction

Human blood pressure is mainly controlled by the renin–angiotensin system, which consists of several key components, including renin, angiotensinogen (AGT),^3^ angiotensin-converting enzyme (ACE), and angiotensin receptors (1). Renin is a highly specific aspartic protease that cleaves AGT between Leu^10^ and Val^11^ to release the N-terminal angiotensin I peptide (1). This peptide is subsequently processed by ACE to form angiotensin II, which is the major biologically active hormone and functions through binding to its receptors. Cleavage of AGT by renin is the rate-limiting step of the renin–angiotensin system (2). This step is tightly regulated in mammals to maintain normal blood pressure, with subtle changes often associated with hypertension and other cardiovascular diseases. For example, a mild increase (10–20%) in plasma concentrations of human AGT caused by the M235T polymorphism is often associated with essential hypertension (3, 4). Also, an AGT mutation where Leu^10^ is replaced by a Phe, resulting in a mere 2-fold increase in the cleavage efficiency of the mutant by renin, is associated with the development of preeclampsia, a life-threatening hypertensive disorder during pregnancy (5).

Previous structural and biochemical studies of renin–AGT interactions have revealed that renin specifically recognizes and cleaves the N-terminal tetradecapeptide of AGT, the only known natural substrate of renin. It has also been shown that the body of the AGT molecule is critical for efficient angiotensin I release by renin, as the *K_m_* value for AGT is 10-fold lower than that of a synthetic 14-residue peptide derived from the AGT N terminus (6, 7). Our previous structural characterizations of AGT and renin have shown that AGT adopts the typical serpin (serine protease inhibitor) framework with a central β-sheet and a surface-exposed reactive loop and that binding of AGT by renin induces substantial movements of both a surface loop and the N-terminal tail, linked by a labile disulfide bond (8). However, due to limited resolution (4.35 Å) of the AGT–renin complex structure, the detailed molecular interactions between AGT and renin remain obscure. Furthermore, human plasma AGT is a heterogeneous glycoprotein resulting from its variable glycosylation. One of the glycosylation sites at Asn^14^ is close to the scissile bond (Leu^10^–Val^11^) and has been shown to affect the efficiency of angiotensin I release (9, 10). It is unclear how the presence of carbohydrate at the glycosylation sites would affect the conformation and activity of AGT.

To address these questions, here we have solved high-resolution crystal structures of human glycosylated AGT, its encounter complex with renin, AGT cleaved in the reactive center loop, and the spent AGT from which the N-terminal angiotensin peptide has been removed by renin. These structures together with detailed biochemical characterizations revealed that, when renin binds AGT, the N-terminal angiotensin tail is inserted into a pocket on AGT that is a hormone-binding site in other serpins. This and other binding interactions induce profound conformational changes in AGT.

## Results

### Crystal structures of human glycosylated AGT, its complex with renin, loop-cleaved AGT, and spent AGT

To understand how renin binds AGT and how glycosylation affects their interaction, here we have prepared glycosylated human AGT variants (N137Q/N271Q/N295Q/C232S/C308S, termed AGT-N14) where only a single glycosylation site on Asn^14^ is retained. Subsequently, we prepared spent AGT, where the N-terminal 10-residue angiotensin I peptide was removed from the AGT-N14 expression construct and loop-cleaved AGT, where AGT-N14 was cleaved between Gln^412^ and Leu^413^ by thermolysin treatment. An inactive human renin variant (D226A) was also prepared from HEK293 cells for crystallizing the encounter complex of renin and AGT. The structures of AGT, its complex with renin, the spent AGT, and RCL-cleaved AGT were solved at 2.3, 2.55, 2.6, and 2.97 Å resolution, respectively. All structures were refined to good geometry with statistics shown in Table 1.

**Table 1:** **Data collection and refinement statistics**

	Intact human AGT	AGT–renin complex	Spent AGT	RCL-cleaved AGT
**Residue ranges*^a^***	34–485	AGT: 34–485	44–485	34–485
		Renin: 67–406		

**Data collection**				
Synchrotron stations	DLS I04-1	DLS I04-1	DLS I04	DLS I02
Space group	P4_1_	P6_3_22	P2	P4_1_
*a*, *b*, *c* (Å)	71.35, 71.35, 125.3	124.1, 124.1, 260.9	86.3, 41.04, 129.95	80.14, 80.14, 117.13
α, β, γ (degees)	90, 90, 90	90, 90, 120	90, 107.82, 90	90, 90, 90
Resolution (Å)*^b^*	46.8–2.3 (2.38–2.3)	62.06–2.55 (2.65–2.55)	43.44–2.63 (2.7–2.63)	66.14–2.97 (3.05–2.97)
*R*_merge_*^b^*	0.057 (0.702)	0.209 (1.251)	0.139 (0.801)	0.214 (1.282)
〈I/σ(I)〉*^b^*	16.6 (2.3)	10.2 (2.1)	6.9 (1.3)	6.5 (1.4)
CC_½_*^b^*	0.999 (0.511)	0.998 (0.674)	0.987 (0.534)	0.987 (0.530)
Completeness (%)*^b^*	99.9 (99.9)	100 (100)	99.3 (99.3)	100 (100)
Redundancy*^b^*	6.5 (5.4)	12.7 (12.9)	3.3 (3.3)	6.7 (7.1)

**Refinement**				
*R*_work_/*R*_free_	0.178/0.196	0.208/0.234	0.241/0.284	0.219/0.237
No. of unique reflections*^b^*	27,786 (2704)	39,584 (4393)	26,230 (1930)	14,563 (1106)
No. of atoms (non-hydrogen)	3328	6136	5923	3336
Average *B*-factors	63.5	40.4	42.5	68.7
r.m.s. deviations				
Bond lengths (Å)	0.003	0.002	0.003	0.003
Bond angles (degrees)	0.613	1.192	0.707	0.754
Ramachandran favored region (%)	96.8	97.6	97.7	96.4
Ramachandran outliers (%)	0	0	0	0
MolProbity score*^c^*	1.54 (99%)	0.85 (100%)	1.28 (100%)	1.27 (100%)

**PDB code**	5M3Y	6I3F	5M3X	6I3I

*^a^* Corresponding to UniProt entry P01019 for AGT and P00797 for renin.

*^b^* Values in parentheses are for highest-resolution shell.

*^c^* 100th percentile is the best among structures of comparable resolutions. 0th percentile is the worst.

### The N-terminal tail is sequestered in native AGT

**Figure 1.**
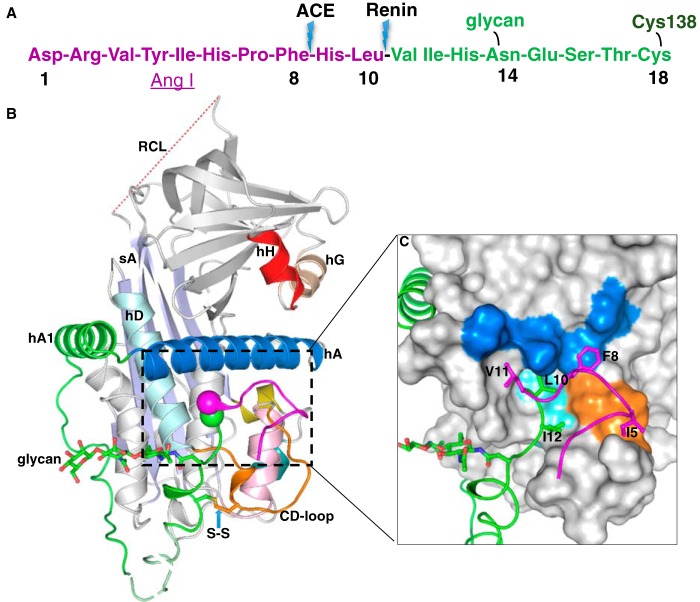
**The crystal structure of human glycosylated AGT.** *A*, the N-terminal tail sequence of AGT, indicating the renin and ACE cleavage sites, the glycosylation site, and the conserved disulfide bond. *B*, the structure of AGT is shown as a *cartoon*. The serpin template is in *gray*, and helix A (*hA*) is in *marine* with the A-sheet (*sA*) in *light blue* and the disordered RCL in *red dashes*. The Ang I segment is in *magenta*, and the following amino-tail is in *green* with the scissile bond shown as *magenta* and *green spheres*. Cysteine 18 in the amino tail forms a disulfide bond with cysteine 138 of the CD-loop (*brown*). The glycan attached to Asn^14^ is shown as *green sticks*. The segment from Glu^20^ to Pro^29^ (*dashed*, *pale green*) is disordered in the structure and modeled for illustration. Helix H (*hH*) is in *red*, and helix G (*hG*) is in *wheat color. C*, *surface representation* of the main body of AGT, with the extra N terminus (residues 1–63) shown in a *cartoon representation*. The Ang I peptide is mainly stabilized by hydrophobic interactions with the main body. Residues Ile^5^, Phe^8^, Leu^10^, Val^11^, and Ile^12^ (shown as *sticks*) form hydrophobic interactions with residues in the CD-loop (Val^131^, Pro^132^, and Trp^133^ as *brown surface*), helix A (Leu^68^, Met^72^, Leu^76^, and Phe^79^ as *marine surface*), and helix D (*hD*; Leu^142^ and Val^147^ as *cyan surface*). The scissile bond (Leu^10^–Val^11^) is buried in the hydrophobic cavity.

The crystal structure of AGT-N14 shows that AGT retains a typical serpin fold with the extra 63-residue extension at the N terminus being well-ordered, similar to the previous unglycosylated AGT structures (8). The conserved disulfide bond between Cys^18^ and Cys^138^ is properly formed, and the N-terminal tail residues form extensive hydrophobic interactions and several hydrogen bonds with the body of AGT (Fig. 1, *A* and *B*). Residues Ile^5^, Phe^8^, Leu^10^, Val^11^, and Ile^12^ pack with residues (Leu^68^, Met^72^, Leu^76^, and Phe^79^) from helix A and residues (Val^131^, Trp^133^, Leu^142^, and Val^147^) from the CD loop (loop connecting helices C and D) and helix D. This leads to the scissile bond of Leu^10^–Val^11^ being buried in an inaccessible position (Fig. 1*C*). The potential drawback of this configuration is that AGT has to undergo conformational changes for the scissile bond to move out to bind renin, whereas the advantage is that it could protect the scissile bond from nonspecific cleavage by other proteases.

### The binding interactions between renin and AGT

**Figure 2.**
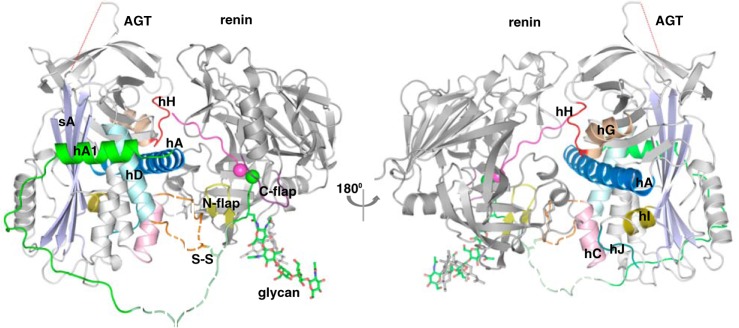
**The crystal structure of the AGT–renin complex.** The *color scheme* of the AGT moiety in the complex is the same as in Fig. 1, whereas the renin moiety is shown as *gray* with *yellow* N-flap and *purple* C-flap. The renin molecule is in contact with the surface of AGT containing helix H (*hH*), helix A (*hA*), helix C (*hC*), and the CD-loop. The scissile bond (shown as *spheres*) is located in the renin active cleft. The fragments without clear electron density are shown with *dashed lines* for illustration.

**Figure 3.**
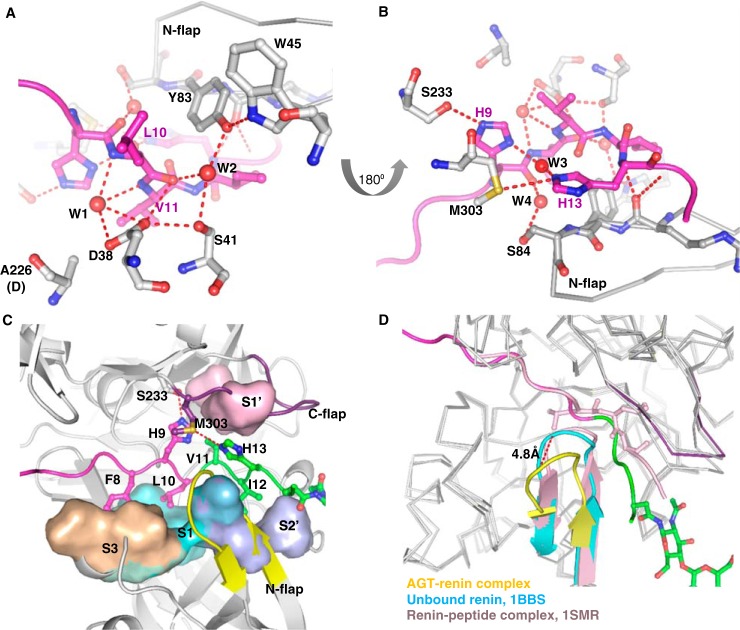
**Interactions inside the renin active cleft.** *A*, renin residues are shown as *gray*, and the AGT peptide is in *magenta*. Asp^38^ in renin is hydrogen-bonded to the carbonyl oxygen of the scissile bond and a water (*W1*) that is also within hydrogen-bond range to the other mutated aspartic acid (Ala^226^). The conserved hydrogen bond network Trp^45^-Tyr^83^-water (W2)-Ser^41^-Asp^38^ is present. *B*, His^9^ in AGT is hydrogen-bonded to Ser^233^. His^9^ also connects to His^13^ through a bridging water (*W3*). Ser^84^ in the N-flap forms a hydrogen bond with a water (*W4*), which connects to the carbonyl oxygen of His^9^. *C*, binding subsites in the renin active cleft. Subsite S3 (Pro^118^, Phe^119^, Leu^121^, Ala^122^, and Phe^124^ of renin) accommodates Phe^8^; S1 (Phe^119^, Phe^124^, Val^127^, Val^36^, and Tyr^83^) accommodates Leu^10^; S1′ (Leu^224^ and Ile^305^) accommodates Val^11^; and S2′ (Ile^137^, Leu^81^, and Tyr^83^) accommodates Ile^12^. The N-flap of renin is shown as *yellow*, and the C-flap is in *purple. D*, the N-flap of renin (*yellow*) in the AGT–renin complex adopts an “open” position with a movement of 4.8 Å compared with its conformation in the unbound renin (*cyan*, PDB code 1BBS). There is no such conformational change when a decapeptide substrate (corresponding to residues 4–14 of rat AGT) is bound to mouse submaxillary renin (*pink*; PDB entry 1SMR). The N-terminal residues 1–10 of the AGT peptide are in *magenta* and the following residues 11–15 and the glycan are shown as *green*.

The crystal structure of the AGT–renin complex shows that the N-terminal tail of AGT is docked in the substrate-binding pocket of renin (Fig. 2). The total surface area buried in the interface between AGT and renin is more than 2100 Å^2^; interactions between the N-terminal peptide and the renin active site account for about 50% of the buried surface, whereas interactions between the bodies of AGT and renin account for the rest. Renin is an aspartic protease in which the active site is formed by the junction of two similar domains, each containing an aspartic acid residue (Asp^38^ and Asp^226^ in human renin) to form a catalytic dyad that hydrolyzes the peptide bond (11). Although the Asp^226^ of renin was mutated to alanine to obtain a stable initiation complex, the binding interactions within the renin active site largely resemble those seen in the crystal structure of a renin–peptide complex solved previously (PDB entry 1SMR) (12). The Asp^38^ of renin is hydrogen-bonded to the carbonyl oxygen of the scissile bond and to a water molecule that is also within hydrogen-bond distance to the other mutated aspartic acid (Fig. 3*A*). Therefore, the active site conformation in our structure is consistent with the well-accepted proposal (13) that Asp^38^ protonates the carbonyl oxygen of the scissile bond of the natural protein substrate, whereas the Asp^226^ carboxyl is ionized and deprotonates the catalytic water molecule that initially bridges the two aspartates. One of the imidazole nitrogens (NE2) of His^9^ is hydrogen-bonded to the Oγ of Ser^233^, whereas the other nitrogen (ND1) connects His^13^ through a bridging water (Fig. 3*B*). Neither His^9^ nor His^13^ are connected to the catalytic dyad directly or indirectly. Therefore, it is unlikely that the peak at the slightly acidic pH 6.5 in the pH activity profile for the cleavage of AGT by human renin is solely determined by the histidines around the scissile bond (12, 14, 15). The hydrogen bond network Trp^45^-Tyr^83^-water-Ser^41^-Asp^38^ is also present, connecting the N-flap of renin with the catalytic site (Fig. 3*A*). Such a network is conserved in aspartic proteases and has been shown to be essential for renin activity (16). However, there are subtle difference in the renin conformations between the complexes. The N-flap of renin in the renin–AGT complex is in a relatively open conformation with the tip of the flap shifted by about 5 Å when compared with that seen in the renin–peptide complex (Fig. 3*D*). This is likely induced by the longer N-terminal tail of AGT, which holds renin against the body of AGT. In addition to the essential hydrogen bonds around the active site, hydrophobic interactions account for the complementary steric fitting of the peptide in the renin-binding pocket. There are four main subsites: S3, S1, S1′, and S2′ (according to the nomenclature of Schechter and Berger (17)), accommodating Phe^8^, Leu^10^, Val^11^, and Ile^12^ of the peptide (Fig. 3*C*). It has been suggested that the sizes and shapes of these binding pockets partly determine the species specificity of the action of renin on AGT (12).

**Figure 4.**
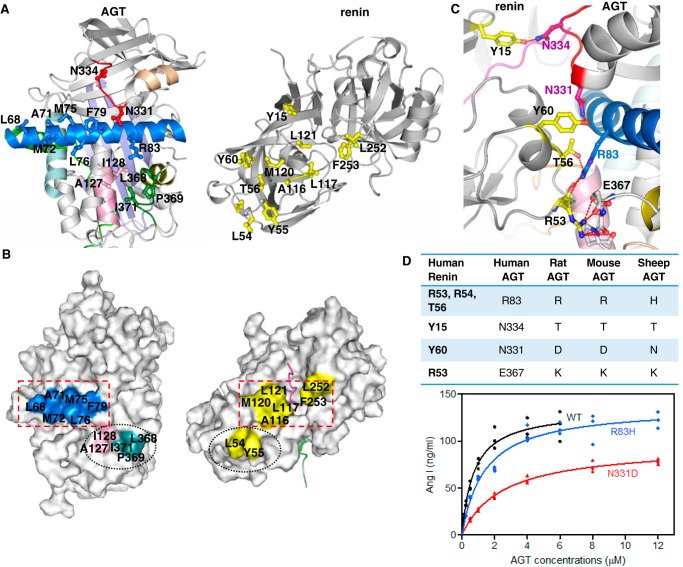
**Interactions between renin and angiotensinogen in the complex.** *A*, an *opened-up cartoon view* of the interface (with the renin moiety rotated 180° around the *y* axis) illustrating the hydrophobic interactions and hydrogen-bonded residues (*sticks*). *B*, an *opened-up surface representation* highlights hydrophobic interactions at the interface. *C*, *cartoon view* of the interface, with hydrogen bonds shown as *red dashes* between interacting residues (*sticks*). Renin, on the *left*, is shown as *gray* with interacting residues in *yellow*. AGT, on the *right*, is shown using the *colors* defined for Fig. 1. *D*, residues involved in hydrogen-bonding interactions are listed, along with the identities of corresponding residues in AGT from other species. The plot shows that cleavage efficiency is reduced when Asn^331^ or Arg^83^ of human AGT is replaced by an equivalent residue from rat or mouse AGT, respectively. The detailed kinetic constants are listed in Table 2.

The substantial interactions between the bodies of the two molecules are mainly hydrophobic (Fig. 4, *A* and *B*). The surface of AGT involved in binding renin includes residues from helix H, helix A, helix C, the CD-loop, and the IJ-loop (loop connecting helices I and J). Helix A of AGT lies across the binding interface with side chains of six hydrophobic residues (Leu^68^, Ala^71^, Met^72^, Met^75^, Leu^76^, and Phe^79^) facing toward renin and stacking with Ala^116^, Leu^117^, Met^120^, Leu^121^, Leu^252^, and Phe^253^ of renin (Fig. 4*B*). Residues in helix C (Ala^127^ and Ile^128^) and in the IJ-loop (Leu^368^, Pro^369^, and Ile^371^) also form hydrophobic interactions with renin residues Leu^54^ and Tyr^55^. There are also several hydrogen bonds between the bodies of the two molecules (Fig. 4*C*). Arg^83^ of AGT forms a cluster of hydrogen bonds with renin residues (Arg^53^, Leu^54^, and Thr^56^). Asn^331^ and Asn^334^ in the unwound helix H form hydrogen bonds with Tyr^60^ and Tyr^15^ of renin, respectively. Arg^53^ in renin forms a salt bridge with Glu^367^ and a cluster of hydrogen bonds with the main chain of the IJ-loop in AGT.

It has been well-documented that human renin cannot efficiently process AGT from mouse and rat (18–20). A sequence alignment shows that, of the interacting residues, Arg^83^, Asn^331^, and Asn^334^ are not strictly conserved among species. To assess the importance of these residues in AGT, we prepared AGT mutants in which Arg^83^, Asn^331^, and Asn^334^ were replaced with the corresponding residues from rat, mouse, and sheep AGT and assessed their binding affinity and catalytic efficiency toward human renin (Fig. 4*D*, Table 2, and Fig. S1). The N334T mutation did not significantly affect either the binding affinity or the catalytic efficiency. The catalytic efficiency for cleavage of AGT R83H was about half that of the WT, and the binding affinity was 2.2-fold lower. Most significantly, the binding affinity of the N331D variant decreased about 4.6-fold with the consequence of a 5-fold decrease in the catalytic release of angiotensin I. This indicates that residues involved in the body-to-body interface between AGT and renin play a key role in determining the catalytic efficiency and binding affinity of renin. This explains the species specificity of human renin toward AGT from other mammals and also explains why intact AGT has a 10-fold lower *K_m_* value compared with the tetradecapeptide derived from its N terminus (6, 7).

**Table 2:** **Binding affinity and kinetic parameters of human renin–angiotensinogen interaction** Bio-layer interferometry was used to measure the binding kinetic constants (*k*_on_, *k*_off_, and *K_D_*) between the inactivated human renin mutant (D226A) and human AGT in both WT and mutant forms (as indicated in the left column; N4 represents the quadruple glycosylation mutant N14Q/N137Q/N271Q/N295Q). The enzymatic kinetic parameters (*K_m_*, *k*_cat_, and *k*_cat_/*K_m_*) for the cleavage efficiency by WT human renin of human AGT mutants (the same panel of AGT mutants as the binding assay) were measured by quantifying the released Ang I. All of the proteins were expressed in HEK293EBNA cells. The mean and S.D. were computed from three independent experiments.

AGT	*k*_on_	*k*_off_	*K*D	*k*_cat_	*K*m	*k*_cat_/*K*m
	*1/ms*	*1/s*	*nm*	*s*^−*1*^	μ*m*	*s*^−*1*^ μ*m*^−*1*^
WT	2.15 ± 0.36 × 10^4^	4.37 ± 1.2 × 10^−4^	20.3 ± 2.2 (100%)	0.20 ± 0.01	0.76 ± 0.08	0.25 ± 0.01 (100%)
N331D	3.21 ± 0.81 × 10^3^	2.96 ± 0.88 × 10^−4^	91.5 ± 5.3 (456%)	0.14 ± 0.01	2.56 ± 0.37	0.05 ± 0.01 (21%)
R83H	1.40 ± 0.27 × 10^4^	6.06 ± 0.37 × 10^−4^	44.6 ± 11.1 (222%)	0.20 ± 0.03	1.42 ± 0.12	0.14 ± 0.01 (54%)
N334T	1.50 ± 0.01 × 10^4^	4.18 ± 0.56 × 10^−4^	27.9 ± 3.7 (138%)	0.16 ± 0.01	0.76 ± 0.05	0.22 ± 0.01 (84%)
N4	7.46 ± 1.57 × 10^4^	8.61 ± 0.49 × 10^−5^	1.2 ± 0.2 (5.9%)	0.44 ± 0.02	0.67 ± 0.10	0.65 ± 0.08 (255%)
N14Q	3.61 ± 0.04 × 10^4^	2.09 ± 0.39 × 10^−4^	5.8 ± 1.1 (28.9%)	0.35 ± 0.01	0.56 ± 0.02	0.64 ± 0.03 (252%)

To further characterize the binding interactions between the bodies of AGT and renin, we prepared spent AGT where the angiotensin peptide is removed by renin, measured its binding affinity toward renin, and tested its effect on renin cleavage of intact AGT. One might expect spent AGT to have modest binding affinity toward renin, as the binding interface between the bodies of two molecules covers more than 1000 Å^2^ of buried surface; however, we found that spent glycosylated AGT cannot form a stable complex with renin when analyzed by gel filtration or native PAGE (data not shown). The binding affinity derived from steady-state analysis gives a *K_D_* value of 13 μm (Fig. S2), which is 640-fold weaker than the binding of intact AGT to renin. We then tested whether spent AGT could interfere with the interaction between renin and intact AGT through a product inhibition effect. As shown in Fig. S3, renin readily cleaves an AGT fusion protein to generate spent AGT. When increasing amounts of spent AGT were added, substantial inhibition arose at a concentration of 0.65 μm spent AGT (Fig. S3, *lane 5*), which is about half the concentration of intact AGT in the assay (1.1 μm). As a negative control, the addition of another serpin, protein Z–dependent inhibitor, had no effect on renin cleavage of AGT. The concentration of circulating intact AGT in plasma of normotensive subjects is about 1.1 μm, and the spent AGT appears to be far lower (21, 22). Although the relative abundance of spent AGT *versus* intact AGT increases in plasma in some pathologic states when renin secretion is stimulated (21), it is still highly unlikely that the spent AGT concentration could be on the same level as the intact AGT. Therefore, we conclude that spent AGT does have a product inhibition effect on renin cleavage of intact AGT; however, it is unlikely to be significant *in vivo*. It appears that the optimal binding affinity between the bodies of AGT and renin has been selected to enhance the activity of renin and at the same time to avoid significant product inhibition under physiological conditions. The lack of ordered structure for residues 16–28 near the N terminus or for residues 132–141 in the CD-loop in bound AGT may be relevant; if these residues were well-ordered, they would be positioned to interact with the N-flap of renin, which could increase binding affinity beyond the optimal range.

### Conformational changes of human AGT induced by renin binding

**Figure 5.**
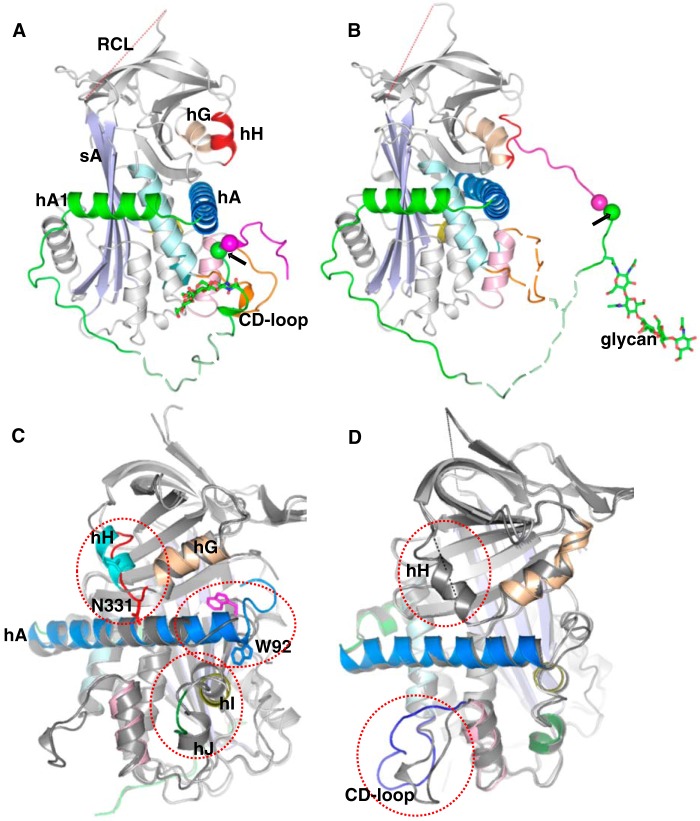
**Conformational changes of angiotensinogen upon renin interaction.** *Side views* of AGT alone (*A*) and from the complex with renin (*B*) illustrate the substantial movement of the N terminus of AGT into the renin active cleft. The scissile bond (shown as *spheres*) moves 18.6 Å during the complex formation. The *color coding* and abbreviations are the same as in Fig. 1. *C*, superposed structures of native AGT and AGT from the complex with renin show significant structural rearrangement of helices H, A, I, and J (*hH*, *hA*, *hI*, and *hJ*) of AGT upon renin binding. Native AGT is shown as *gray* with a cyan helix H, and AGT in the complex is *color-coded* as AGT in Fig. 1. Helix H is completely unwound with the C^α^ atom of Asn^331^ (shown as a *cyan stick* in native AGT and *red stick* in AGT complexed with renin) shifting more than 6 Å to interact with Tyr^60^ of renin. Helix A is extended by two turns with the side chain of Trp^92^ (shown as a *magenta stick* in native AGT and a *marine stick* in AGT complexed with renin) at the tip of helix A being flipped out. *D*, superposed structures of native (*gray*) and spent AGT (same *color coding* as AGT in Fig. 1) show that spent AGT largely resembles native AGT, but with differences in helix H and the CD-loop (*blue* in spent AGT). Helix H of spent AGT is completely disordered.

Comparison of structures of native AGT and its complex with renin shows that the N-terminal tail of AGT undergoes significant conformational changes to dock into the substrate binding site of renin with the scissile bond presented appropriately for hydrolysis by the two active aspartate residues (Fig. 5, *A* and *B*). In native AGT, the region of the N-terminal tail near the scissile bond Leu^10^–Val^11^ forms hydrophobic interactions with helix A residues, such as Leu^68^, Met^72^, Leu^76^, and Phe^79^ (Fig. 1*C*). These interactions are lost on binding to renin, when the scissile bond shifts about 19 Å from its sequestered position to an extended configuration (Fig. 5, *A* and *B*) but are replaced by new interactions with renin residues, such as Leu^117^, Leu^121^, Leu^252^, and Phe^253^ (Fig. 4*B*). Complex formation is also associated with secondary structural changes in other areas of AGT. Notably, helix A is extended by nearly 2 turns and bent toward the core of AGT, with the side chain of Trp^92^ at the C-terminal part of helix A flipping out to allow space (Fig. 5*C*). Minor conformational changes around helices I and J are also likely induced by renin binding.

**Figure 6.**
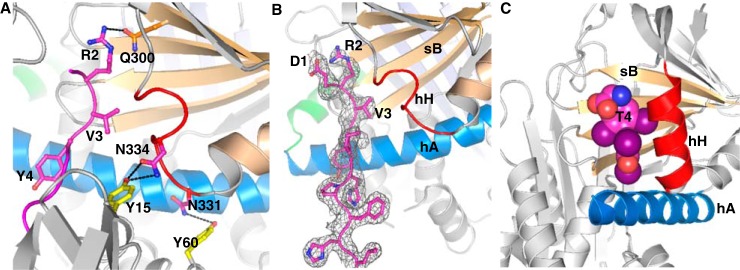
**The insertion of the N-terminal peptide into the hormone binding pocket.** *A*, renin binding results in the N terminus of AGT being inserted into the hormone binding pocket and in unwinding of helix H. The side chain of Arg^2^ is largely buried and forms stabilizing interactions with Gln^300^. Val^3^ and Tyr^4^ also form hydrophobic interactions with surrounding residues. *B*, there is clear electron density for all of the residues in the N terminus (*gray mesh*, 2*mF_o_* − *DF_c_* map, contoured at 1.0σ, including density within 1.6 Å of atoms in the N terminus). *C*, a similarly located pocket is used to bind thyroxine (*T4*; *spheres*) in TBG.

Most unexpectedly, we have found that the N terminus of AGT emerges from the renin active cleft and pokes into a surface cavity surrounded by helix H, sheet B, and helix A of AGT (Fig. 6, *A* and *B*). This cavity corresponds to the hormone-binding pocket seen in two other serpins, thyroxine-binding globulin (TBG; Fig. 6*C*) and cortisol-binding globulin (CBG) (23). The side chain of Arg^2^ of the N terminus is completely buried in the relatively shallow pocket and is stabilized by hydrogen bonds with neighboring residue Gln^300^ (Fig. 6*A*). The side chain of Val^3^ interacts with hydrophobic residues within this hormone-binding pocket, including Met^336^. The entire helix H (residues 330–336) is unwound, with the shift of residues 333–337 opening up the pocket to accommodate the N-terminal tail and with Asn^331^ and Asn^334^ shifting to form key hydrogen bonds with the body of renin (Figs. 4*C* and 5*C* and Table 2). At the same time, helix G is extended by a half-turn. AGT appears to have adopted a “tail-in-mouth” mechanism for interacting with renin, where insertion of its N terminus into the hormone-binding pocket helps to stabilize a new conformation that forms a complementary surface on AGT for renin binding. It is tempting to speculate that any ligands that bind tightly to this pocket in intact AGT would attenuate the interaction between AGT and renin and have an anti-hypertensive effect.

To further dissect the conformational plasticity of AGT, we then solved the crystal structure of spent AGT and compared it with that of intact AGT. The spent AGT retains a fold similar to that of native AGT with an r.m.s. deviation of 0.63 Å over 355 Cα atoms (Fig. 5*D*). Helices A, I, and J of spent AGT, which are involved in renin binding, have the same conformation as in native AGT. Hence, it is possible that the native and spent AGT have similar angiotensin I–independent functions through hypothetical receptors to the same serpin characteristic binding sites (24, 25). In contrast, there are significant movements in the CD-loop. To some extent, this may reflect flexibility in this part of the molecule, influenced by different crystal-packing environments; the CD-loop is involved in packing interactions in the crystals of both intact and spent AGT. However, it is notable that the rearrangement of the CD-loop in spent AGT places the side chain of Trp^133^ in the hydrophobic pocket occupied by the scissile peptide region in intact AGT, replacing the side chain of Val^11^. Finally, helix H in the spent human AGT is disordered with no clear electron density, whereas it is in a helical conformation in intact AGT. This may also be influenced by crystal packing; if helix H in spent AGT were ordered in the same conformation as in intact AGT, it would clash with the CD-loop of a neighboring molecule. The lability of helix H in human AGT is reminiscent of the rat and mouse AGT structures (PDB codes 2WXX, 2WXY, 2WXZ, 2WY0, and 2WY1), in all of which helix H exists in an unwound or slightly distorted conformation. It is plausible that helix H of AGT is inherently unstable and can equilibrate between both conformations, only forming favorable interactions with renin when induced by the insertion of the N terminus of AGT in the hormone-binding pocket. A reduced stability of helix H in spent AGT could partly explain why spent AGT only has a relatively low binding affinity toward renin (Fig. S2).

To confirm conclusions from biochemical experiments that cleavage of the reactive center loop of AGT does not trigger the stressed-to-relaxed (S-to-R) transition characteristic of inhibitory serpins, we solved the structure of AGT cleaved by thermolysin treatment and compared it with that of intact AGT. Loop cleavage indeed induces no significant conformational changes, with an r.m.s. deviation of only 0.28 Å over 371 C^α^ atoms (Fig. S4). The cleaved loop is disordered, but is not inserted into the central β-sheet A.

### Glycosylation effect on AGT

It has been demonstrated that glycosylation of human AGT is not crucial for its intracellular trafficking and secretion, but it does play an important role in affecting the interactions between AGT and renin with altered enzyme kinetic parameters (9). Notably, unglycosylated AGT is a better substrate than glycosylated AGT for renin cleavage, and the carbohydrate at glycosylation site Asn^14^ seems to play a key role in affecting renin activity (5, 22). It is further confirmed here that both the quadruple glycosylation mutant N14Q/N137Q/N271Q/N295Q, termed N4, where all four glycosylation sites were mutated, and the AGT N14Q variant can be processed by renin about 2.5-fold faster than the fully glycosylated WT AGT (Table 2). Here in our crystal structures, glycans linked to glycosylation site Asn^14^ could be resolved with clear electron density (Fig. S5). In the native AGT structure, the oligosaccharide attached to Asn^14^ forms several stabilizing hydrogen bonds to the surrounding residues of AGT (Lys^146^, Ser^16^, and Thr^17^) (Fig. S5*A*), whereas in the complex structure, the carbohydrates make no direct contacts with neighboring renin residues (Fig. S5*B*). As the Cα atom of Asn^14^ shifts about 16 Å upon complex formation, it is likely that shifting the bulky carbohydrates linked to Asn^14^ and breaking their hydrogen bonds during renin binding contribute to the lower efficiency of renin in processing glycosylated AGT. Thus, this glycosylation at Asn^14^ probably also contributes to the well-known observation that human AGT can only be efficiently cleaved by primate renin but not by renins from nonprimates that lack Asn^14^ in their copies of AGT (9, 10, 26).

## Discussion

The serpin superfamily is classically characterized by proteins that fold into a conserved metastable tertiary structure that has the ability to undergo profound conformational changes for protease inhibition. However, three members of this superfamily, TBG, CBG, and AGT, have lost the ability to inhibit proteases and become hormone carriers instead. TBG and CBG are close homologues, with thyroxine and cortisol binding to equivalent pockets on the serpin framework, formed by helices H and A and strands 3–5 of the B-sheet (23, 27, 28). Thyroxine and cortisol release are triggered by a conformational S-to-R transition similar to that seen in inhibitory serpins. In contrast, it has been shown that AGT has lost the ability to undergo this typical serpin S-to-R transition (29), confirmed here by our structure of loop-cleaved AGT, so it was very puzzling why the serpin framework was selected in the course of evolution as an angiotensin carrier. Does the serpin structure of AGT merely provide a carrier for the added-on tail? One part of the answer may be that AGT was originally a bifunctional protein serving both as a protease inhibitor and the donor of a peptide hormone, which still seems to be the case in the lamprey (30–32).

Here, we have addressed this puzzle by solving the structures of glycosylated AGT and its complex with renin. This reveals for the first time the extensive hydrophobic interactions and hydrogen bonds in the renin active site and in the interface between the two components. Renin binding of AGT requires substantial conformational changes in the N-terminal tail of AGT, in which the scissile peptide of angiotensin is expelled from a sequestered position to dock into the active-site pocket of renin. This mode of renin binding is likely achieved through competition between the N-terminal tail of AGT and the body of renin for binding to helix A of AGT. Most remarkably, we found that the N terminus of AGT inserts into the equivalent of the hormone-binding pocket seen in TBG and CBG. This insertion is accompanied by the unwinding of helix H, which we know from structures of rat and mouse AGT to be inherently unstable; this unwinding allows residues such as Asn^331^ and Asn^334^ to form critical interactions with renin. The conformational change shifts the Cα atom of Asn^331^ by more than 6 Å during complex formation. Substituting this residue with the corresponding Asp^331^ seen in mouse or rat AGT results in a 5-fold decrease in catalytic efficiency of renin cleavage (Table 2), consistent with the finding that human renin cleaves mouse and rat AGT with significantly lower catalytic efficiency than human substrate. Thus, renin binding of AGT induces concerted movements in angiotensin, including expulsion of the N-terminal tail from a sequestered position, docking into the renin active site, insertion of the N terminus into the hormone-binding pocket, unwinding of helix H, and subsequent extension of helix G, all of which ultimately leads to a complementary binding interface between the bodies of AGT and renin. Altogether, this further supports the idea that AGT is not just a passive carrier of an angiotensin peptide and that sophisticated use is made of its serpin framework, especially its “hormone-binding” pocket, in the efficient release of angiotensin by renin cleavage.

The serpin framework, like many other protein scaffolds, is also subject to post-translational modifications such as oxidation or glycosylation, and these modifications can have significant effects on activity or conformation. For example, the activity of SerpinB9, a regulator of the cytotoxic lymphocyte granzyme B, is reversibly inhibited by vicinal disulfide bond formation in the reactive loop (33). In our previous study (8), we revealed that AGT has a conserved, labile disulfide bond between Cys^18^ and Cys^138^ and that oxidized AGT is a better substrate than the reduced form for renin cleavage. Most intriguingly, it was found that an increased ratio of oxidized AGT over the reduced form in plasma is associated with preeclampsia, a hypertensive disease during pregnancy (8). This finding has now been confirmed by an independent large case–control study in patients with preeclampsia using an improved ELISA method (34).

Serpin activity can also be modulated by glycosylation. For example, the carbohydrate linked to Asn^135^ in antithrombin can affect heparin binding, with fully glycosylated antithrombin (the α form) having lower affinity (35). Glycans linked to Asn^347^ in the reactive center loop of CBG also retard proteolysis and subsequent cortisol release (36). Similarly, the presence in AGT of glycans linked to Asn^14^ decrease angiotensin I release by renin cleavage. We speculate that the heterogeneity of glycosylation at this site could serve as an additional control point to modulate angiotensin I release, in the same way that variable glycosylation affects the rate of activation of plasminogen by tissue-type plasminogen activator (37).

As the human renin–angiotensin system differs significantly from those of other species (38) and many residues in the renin–AGT interface are not conserved, we have looked for polymorphisms of these residues. A recent analysis of exome sequences in over 60,000 individuals, carried out by the Exome Aggregation Consortium (39), allows polymorphisms to be detected at much lower frequency in the human population than previously possible. The database reveals that some mutations in residues that interact in the complex between AGT and renin do occur at low frequency. In AGT, an R83H substitution is seen twice in 121,400 sequence reads (a frequency of 1.65 × 10^−5^); we have shown that this substitution would reduce catalytic efficiency by a factor of 2 (Fig. S1 and Table 2). An R83C substitution occurs at the same frequency, and R83L occurs at a frequency of only 1 in 121,400 (8.24 × 10^−6^). Polymorphisms are also found for two of the renin residues that contact Arg^83^ in AGT (Fig. 4*C* and Table 2). The substitution R53H is seen at a frequency of 3.88 × 10^−5^, and R53C is seen at a frequency of 1.4 × 10^−5^. Perhaps the most interesting polymorphism is a C18R substitution in AGT, which occurs at a frequency of 1.95 × 10^−3^, a level at which about 1 in 250 individuals should be heterozygous. It seems likely that the C18R variant would behave in the same way as reduced WT AGT, resulting in a lowered response to the effects of oxidative stress (8) and a somewhat reduced response to triggers of the RAS. It would be intriguing to learn whether individuals carrying at least one copy of this polymorphism have reduced risks of cardiovascular complications, such as hypertension or preeclampsia, or whether it is disadvantageous under any circumstances.

Overall, our findings indicate that, over the course of evolution, AGT has retained many features found in other serpins, adapting them to modulate angiotensin release by renin cleavage. AGT binds renin through a concerted tail-in-mouth movement using a site that binds hormones in other serpins; this contributes to the unwinding of helix H of AGT when forming key interactions with renin in the complementary binding interface. This new understanding of the interaction of renin and AGT provides the basis for the development of new agents to attenuate angiotensin release through targeting the binding interface or the “hormone-binding” pocket of AGT.

## Experimental procedures

### Recombinant protein expression

The encoding genes for full-length human AGT and prorenin including signal peptide were amplified from I.M.A.G.E clones (Source BioScience, catalog no. 4213559 for human AGT and 5188566 for human prorenin) and inserted into the pCEP4 vector (Invitrogen). The coding sequence of spent AGT was inserted into a modified pCEP4 vector with the antitrypsin signal peptide at its N terminus for secretion. All of the constructs included a His_6_ tag at the C terminus of the gene and were verified by DNA sequencing (Source BioScience) before being transfected transiently into HEK293 EBNA cells using polyethyleneimine (linear, molecular weight 25,000, Polysciences, Inc.). Recombinant proteins were purified from concentrated serum-free expression medium by nickel-affinity (HisTrap, GE Healthcare Life Sciences) and anion-exchange chromatography (Hitrap Q, GE Healthcare) consecutively.

### Mutations in the crystallized AGT-N14 protein

Three of four potentially glycosylated asparagines in human AGT were mutated to glutamines to minimize the glycosylation heterogeneity of AGT for crystallization. Two free cysteines (Cys^232^ and Cys^308^) were replaced with serines to avoid potential aggregation.

The actual mutations in AGT-N14 were N137Q/N271Q/N295Q and C232S/C308S. The same mutations were introduced to spent AGT-N14 for crystallization. A cysteine modification experiment confirmed that the Cys^18^–Cys^138^ disulfide bond is properly formed within these variants (Fig. S6). All of the mutations were introduced by site-directed mutagenesis using the QuikChange kit (Agilent Technologies). All the primers for molecular cloning and mutagenesis are listed in Table S1.

### Preparation of reactive center loop–cleaved AGT

The reactive center loop (RCL) cleaved human AGT was made by thermolysin cleavage of the link between Gln^412^ and Leu^413^ of AGT-N14 and confirmed by SDS-PAGE (Fig. S7) and MS. The ratio of protein/protease ratio and reaction time were optimized by incubating AGT-N14 (singly glycosylated at Asn^14^) with a 2-fold serial dilution of thermolysin for various times. The final digestion condition for large-scale preparation combined 1 mg/ml full-length AGT and thermolysin with a protein/protease ratio of 3200:1 (w/w) at room temperature for 3 h in a buffer containing 10 mm Tris, 0.15 m NaCl, and 10 mm CaCl_2_. The reaction was stopped by the addition of 10 mm EDTA and further purified by ion-exchange chromatography before crystallization.

### Preparation of AGT–renin complex

The D226A mutant of human prorenin, with one of the two active-site aspartate residues mutated to Ala, was incubated with trypsin (Sigma) at an optimized 200:1 molar ratio to remove the prosegment, and then the reaction was stopped by adding 1 mm phenylmethylsulfonyl fluoride. After removal of phenylmethylsulfonyl fluoride and buffer exchange to 10 mm Tris, pH 7.4, and 0.15 m NaCl, the resulting inactive renin D226A was deglycosylated with peptide–*N*-glycosidase F (PNGase F) (New England Biolabs), at an optimized ratio of 1 unit of PNGase F to 1 μg of renin. The deglycosylated renin D226A was further purified by binding to a nickel column to remove PNGase F. The stable AGT–renin complex was made by mixing AGT-N14 and inactive renin D226A mutant at a 1:1.2 molar ratio and purified by size-exclusion chromatography using a HiLoad 16/60 Superdex 200 (GE Healthcare) column equilibrated with 10 mm Tris, pH 7.4, and 0.1 m NaCl. Fractions containing the complex were collected and concentrated to 8 mg/ml for crystallization.

### Crystallization, data collection, and structure determination

The diffraction quality crystals of intact, spent, and RCL-cleaved AGT were grown by microseeding in 96-well sitting drop plates (300 nl of reservoir, 200 nl of 10 mg/ml protein, and 100 nl of seeds) against 80 μl of a range of concentrations of (NH_4_)_2_SO_4_ and different pH buffers. The seeds were prepared using unglycosylated AGT crystals grown in 20% PEG 6000, 1.0 m LiCl, and 0.1 m Tris pH 8.0. As frequently observed in previous examples of cross-seeding with nonidentical proteins (40), the resulting crystals did not share the same crystal packing. A similar method was applied to grow crystals of the complex. Optimized complex crystals were obtained by equilibrating 1 μl of 8 mg/ml protein, 1.5 μl of reservoir, and 0.5 μl of seeds in sitting-drop plates against 1 ml of reservoir solution of 1.74 m (NH_4_)_2_SO_4_ and 0.1 m Tris, pH 7.6. The intact and spent AGT crystals, grown in 96-well sitting drop plates, were cryo-protected by adding 0.5 μl of perfluoropolyether oil (Hampton Research) to the top of the drops. The complex crystals were soaked in cryo-solutions of 2 m Li_2_SO_4_, 1 m (NH_4_)_2_SO_4_ plus 0.1 m Tris, pH 7.6, before harvesting. All of the crystals covered with cryo-solutions were flash-cooled in liquid nitrogen.

Diffraction data were collected at beamlines I04 (spent AGT), I04-1 (intact AGT, AGT–renin complex), and I02 (RCL-cleaved AGT) of the Diamond Synchrotron Light Source and processed with Mosflm (41) and Aimless (42) in the CCP4 program suite. Resolution was decided by CC_½_ > 0.5 and 〈*I*/σ〉 over 1 in the outer-resolution shell (43, 44). The structures of intact, loop-cleaved, and spent AGT were solved by molecular replacement in Phaser (45) by searching with the unglycosylated AGT structure (PDB code 2WXW). The crystals of intact and loop-cleaved AGT each contained one copy in the asymmetric unit, whereas the spent AGT crystal contained two copies. The complex structure was solved by molecular replacement using the unglycosylated AGT structure (PDB code 2WXW), from which the N-terminal residues 1–30 had been trimmed, and renin (PDB code 2BKS) as search models. One copy of the complex was present in the asymmetric unit. Manual model building was carried out in COOT (46), and further refinement was performed in Phenix.refine (47) and Refmac (48). The final models were validated using MolProbity (49), and final refinement statistics for all four structures are shown in Table 1. The carbohydrate structures were built with consideration of the correct core carbohydrate linkages (50). Torsion angles for carbohydrate linkages were validated using the program carp (51). Molecular graphics were prepared using PyMOL (52), and protein–protein interactions were analyzed using PDBePISA (53).

### Enzyme kinetics of the human renin and AGT reaction

For steady-state kinetics, the reaction time and renin concentration were optimized to ensure that product formation was linear with time and that the renin concentration was well below the *K_m_* of the system. Briefly, various concentrations of AGT mutants (eight 2-fold dilutions up to around 10 × *K_m_*) were incubated with 0.2 nm renin at 37 °C for 1 h in a buffer of 10 mm Tris, pH 7.4, 0.15 m NaCl, and 0.25 mg/ml BSA. The reactions were stopped by heating the sample at 98 °C for 5 min. The amount of released Ang I peptide was quantified by immunoassay (S-1188 kit, Peninsula Laboratories, LLC) and plotted against the substrate concentrations. The key consideration was that the measured Ang I concentrations must be within the linear range of the standard curve. The kinetic parameters (*V*_max_ and *K_m_*) for the cleavage of AGT by renin were fit with the standard Michaelis–Menten equation using GraphPad Prism version 5, and *k*_cat_ was calculated as *V*_max_/[*E*]*_t_*. The mean and S.D. calculated from three independent experiments are presented in Table 2.

### The affinity of human AGT binding to renin

The binding kinetics for the interaction between AGT and inactivated renin were measured using a FortéBio Octet Red96 biolayer interferometry system (Menlo Park, CA). Renin D226A was biotinylated preferentially on N-terminal α-amino groups at pH 6.2 using EZ-link-NHS-PEG4-Biotin (Thermo Scientific). Such preferential labeling is achieved by using a reaction pH that is lower than the typical range used for reaction by NHS-ester reagents (54). The optimized biotinylation condition was a 1:0.8 ratio of protein to reagent at 4 °C for 2 h. Nonreacted NHS-PEG4-Biotin was removed by a Zeba spin desalting column (7000 molecular weight cut-off; Thermo Scientific). 2.5 μg/ml biotinylated renin D226A mutant was loaded onto streptavidin biosensors (18-5019, FortéBio). A range of 2-fold dilutions of AGT mutants was prepared in 10 mm Tris, pH 7.4, 0.15 m NaCl, and 1% BSA covering 10 × *K_D_* to 0.1 × *K_D_*. Responses were processed with the FortéBio Data Analysis version 7.0 software. The kinetic association rate constant (*k*_on_) and dissociation rate constant (*k*_off_) were generated using a 1:1 binding model by a global fit without linked *R*_max_. The equilibrium dissociation constant (*K_D_*) was calculated as *k*_off_ divided by *k*_on_. For the reaction of renin and spent AGT, as the on and off rates were very fast and all of the association binding curves reached equilibrium, a steady-state analysis was performed to calculate *K_D_*. Three independent assays were performed to calculate the mean and S.D. (Table 2).

### Alkylation of free sulfhydryl groups of cysteines in AGT

5 μl of 1 mg/ml protein was reduced by 5 μl of 10 mm tris(2-carboxyethyl)phosphine at room temperature for 20 min. Then 10 μl of 20 mm methoxypolyethylene glycol (mean molecular weight of 2000)–maleimide (mPEG2000-mal; Sunbright ME-020MA, NOF Europe) was added to alkylate the free sulfhydryl groups of cysteines. The mixture was incubated at room temperature for 40 min. An aliquot of each sample was also incubated with mPEG2000-mal without reduction by tris(2-carboxyethyl) phosphine. Samples were separated by SDS-PAGE and stained with InstantBlue (Expedeon) to observe the band shift after modification. The reduced disulfide bond reacts with two mPEG2000-mal moieties, causing a 4-kDa shift in SDS-PAGE. Note that the two free cysteines, Cys^232^ and Cys^308^, of WT AGT were mutated to serine in this experiment.

## Author contributions

Y. Y., A. Z., R. W. C., and R. J. R. conceptualization; Y. Y., A. Z., R. W. C., and R. J. R. formal analysis; Y. Y. investigation; Y. Y. writing-original draft; A. Z. and R. J. R. funding acquisition; A. Z., R. W. C., and R. J. R. writing-review and editing; R. J. R. supervision.

## Supplementary Material

Supporting Information

## Footnotes

3 The abbreviations used are:

AGT: angiotensinogen
ACE: angiotensin-converting enzyme
CBG: corticosteroid-binding globulin
mPEG: methoxypolyethylene glycol
PNGase F: peptide–*N*-glycosidase F
RCL: reactive center loop
r.m.s.: root mean square
S-to-R: stressed-to-relaxed
TBG: thyroxine-binding globulin.

## References

[1] Fyhrquist F., and Saijonmaa O. (2008) Renin-angiotensin system revisited. J. Intern. Med. 264, 224–236 10.1111/j.1365-2796.2008.01981.x 18793332 PMC7166930

[2] Crowley S. D., and Coffman T. M. (2012) Recent advances involving the renin-angiotensin system. Exp. Cell Res. 318, 1049–1056 10.1016/j.yexcr.2012.02.023 22410251 PMC3625040

[3] Jeunemaitre X., Soubrier F., Kotelevtsev Y. V., Lifton R. P., Williams C. S., Charru A., Hunt S. C., Hopkins P. N., Williams R. R., Lalouel J.-M., and Corvol P. (1992) Molecular basis of human hypertension: role of angiotensinogen. Cell 71, 169–180 10.1016/0092-8674(92)90275-H 1394429

[4] Ward K., Hata A., Jeunemaitre X., Helin C., Nelson L., Namikawa C., Farrington P. F., Ogasawara M., Suzumori K., and Tomoda S. (1993) A molecular variant of angiotensinogen associated with preeclampsia. Nat. Genet. 4, 59–61 10.1038/ng0593-59 8513325

[5] Inoue I., Rohrwasser A., Helin C., Jeunemaitre X., Crain P., Bohlender J., Lifton R. P., Corvol P., Ward K., and Lalouel J. M. (1995) A mutation of angiotensinogen in a patient with preeclampsia leads to altered kinetics of the renin-angiotensin system. J. Biol. Chem. 270, 11430–11436 10.1074/jbc.270.19.11430 7744780

[6] Cumin F., Le-Nguyen D., Castro B., Menard J., and Corvol P. (1987) Comparative enzymatic studies of human renin acting on pure natural or synthetic substrates. Biochim. Biophys. Acta 913, 10–19 10.1016/0167-4838(87)90226-3 3555621

[7] Burton J., and Quinn T. (1988) The amino-acid residues on the C-terminal side of the cleavage site of angiotensinogen influence the species specificity of reaction with renin. Biochim. Biophys. Acta 952, 8–12 10.1016/0167-4838(88)90095-7 3275468

[8] Zhou A., Carrell R. W., Murphy M. P., Wei Z., Yan Y., Stanley P. L. D., Stein P. E., Broughton Pipkin F., and Read R. J. (2010) A redox switch in angiotensinogen modulates angiotensin release. Nature 468, 108–111 10.1038/nature09505 20927107 PMC3024006

[9] Gimenez-Roqueplo A. P., Célérier J., Lucarelli G., Corvol P., and Jeunemaitre X. (1998) Role of *N*-glycosylation in human angiotensinogen. J. Biol. Chem. 273, 21232–21238 10.1074/jbc.273.33.21232 9694881

[10] Inui Y., Orihashi T., Okada E., Nakagawa T., Ebihara A., Suzuki F., and Nakamura Y. (1998) Effects of glycosylation of the residue at position 14 in ovine angiotensinogen on the human renin reaction. Biosci. Biotechnol. Biochem. 62, 1612–1614 10.1271/bbb.62.1612 9757569

[11] Rahuel J., Priestle J. P., and Grütter M. G. (1991) The crystal structures of recombinant glycosylated human renin alone and in complex with a transition state analog inhibitor. J. Struct. Biol. 107, 227–236 10.1016/1047-8477(91)90048-2 1807356

[12] Dhanaraj V., Dealwis C. G., Frazao C., Badasso M., Sibanda B. L., Tickle I. J., Cooper J. B., Driessen H. P., Newman M., and Aguilar C. (1992) X-ray analyses of peptide-inhibitor complexes define the structural basis of specificity for human and mouse renins. Nature 357, 466–472 10.1038/357466a0 1608447

[13] Brás N. F., Ramos M. J., and Fernandes P. A. (2012) The catalytic mechanism of mouse renin studied with QM/MM calculations. Phys. Chem. Chem. Phys. 14, 12605–12613 10.1039/c2cp41422h 22796659

[14] Nabi A. H., Uddin M. N., Nakagawa T., Orihashi T., Ebihara A., Iwasawa A., Nakamura Y., and Suzuki F. (2005) Roles of His9 (P2 subsite) and His13 (P3′ subsite) in angiotensinogen for catalytic reaction of renin. Int. J. Mol. Med. 16, 103–107 15942685

[15] Iwata H., Nakagawa T., Yoshioka Y., Kagei K., Imada K., Nakane C., Fujita H., Suzuki F., and Nakamura Y. (2008) The coexistence of Ser84 in renin and His13 in angiotensinogen brings a pH profile of two separate peaks to the reaction of human renin and sheep angiotensinogen. Biosci. Biotechnol. Biochem. 72, 179–185 10.1271/bbb.70541 18175911

[16] Andreeva N. S., and Rumsh L. D. (2001) Analysis of crystal structures of aspartic proteinases: on the role of amino acid residues adjacent to the catalytic site of pepsin-like enzymes. Protein Sci. 10, 2439–2450 10.1110/ps.ps.25801 11714911 PMC2374050

[17] Schechter I., and Berger A. (1968) On the active site of proteases. 3. Mapping the active site of papain; specific peptide inhibitors of papain. Biochem. Biophys. Res. Commun. 32, 898–902 10.1016/0006-291X(68)90326-4 5682314

[18] Ganten D., Wagner J., Zeh K., Bader M., Michel J. B., Paul M., Zimmermann F., Ruf P., Hilgenfeldt U., and Ganten U. (1992) Species specificity of renin kinetics in transgenic rats harboring the human renin and angiotensinogen genes. Proc. Natl. Acad. Sci. U.S.A. 89, 7806–7810 10.1073/pnas.89.16.7806 1502199 PMC49800

[19] Fukamizu A., Sugimura K., Takimoto E., Sugiyama F., Seo M. S., Takahashi S., Hatae T., Kajiwara N., Yagami K., and Murakami K. (1993) Chimeric renin-angiotensin system demonstrates sustained increase in blood pressure of transgenic mice carrying both human renin and human angiotensinogen genes. J. Biol. Chem. 268, 11617–11621 8505294

[20] Takahashi S., Fukamizu A., Hasegawa T., Yokoyama M., Nomura T., Katsuki M., and Murakami K. (1991) Expression of the human angiotensinogen gene in transgenic mice and transfected cells. Biochem. Biophys. Res. Commun. 180, 1103–1109 10.1016/S0006-291X(05)81180-5 1659395

[21] Genain C., Bouhnik J., Tewksbury D., Corvol P., and Menard J. (1984) Characterization of plasma and cerebrospinal fluid human angiotensinogen and des-angiotensin I-angiotensinogen by direct radioimmunoassay. J. Clin. Endocrinol. Metab. 59, 478–484 10.1210/jcem-59-3-478 6746861

[22] Barrett J. D., Eggena P., Hidaka H., and Sambhi M. P. (1979) *In vitro* inhibition of renin by human des-angiotensin I renin substrate. J. Clin. Endocrinol. Metab. 48, 96–100 10.1210/jcem-48-1-96 422710

[23] Zhou A., Wei Z., Read R. J., and Carrell R. W. (2006) Structural mechanism for the carriage and release of thyroxine in the blood. Proc. Natl. Acad. Sci. U.S.A. 103, 13321–13326 10.1073/pnas.0604080103 16938877 PMC1557382

[24] Célérier J., Cruz A., Lamandé N., Gasc J. M., and Corvol P. (2002) Angiotensinogen and its cleaved derivatives inhibit angiogenesis. Hypertension 39, 224–228 10.1161/hy0202.103441 11847188

[25] Lu H., Wu C., Howatt D. A., Balakrishnan A., Moorleghen J. J., Chen X., Zhao M., Graham M. J., Mullick A. E., Crooke R. M., Feldman D. L., Cassis L. A., Vander Kooi C. W., and Daugherty A. (2016) Angiotensinogen exerts effects independent of angiotensin II. Arterioscler. Thromb. Vasc. Biol. 36, 256–265 10.1161/ATVBAHA.115.306740 26681751 PMC4732917

[26] Hatae T., Takimoto E., Murakami K., and Fukamizu A. (1994) Comparative studies on species-specific reactivity between renin and angiotensinogen. Mol. Cell. Biochem. 131, 43–47 10.1007/BF01075723 8047064

[27] Qi X., Loiseau F., Chan W. L., Yan Y., Wei Z., Milroy L.-G., Myers R. M., Ley S. V., Read R. J., Carrell R. W., and Zhou A. (2011) Allosteric modulation of hormone release from thyroxine and corticosteroid-binding globulins. J. Biol. Chem. 286, 16163–16173 10.1074/jbc.M110.171082 21325280 PMC3091225

[28] Zhou A., Wei Z., Stanley P. L. D., Read R. J., Stein P. E., and Carrell R. W. (2008) The S-to-R transition of corticosteroid-binding globulin and the mechanism of hormone release. J. Mol. Biol. 380, 244–251 10.1016/j.jmb.2008.05.012 18513745

[29] Stein P. E., Tewkesbury D. A., and Carrell R. W. (1989) Ovalbumin and angiotensinogen lack serpin S-R conformational change. Biochem. J. 262, 103–107 10.1042/bj2620103 2818556 PMC1133235

[30] Fournier D., Luft F. C., Bader M., Ganten D., and Andrade-Navarro M. A. (2012) Emergence and evolution of the renin-angiotensin-aldosterone system. J. Mol. Med. 90, 495–508 10.1007/s00109-012-0894-z 22527880 PMC3354321

[31] Wang Y., and Ragg H. (2011) An unexpected link between angiotensinogen and thrombin. FEBS Lett. 585, 2395–2399 10.1016/j.febslet.2011.06.021 21722639

[32] Wei H., Cai H., Wu J., Wei Z., Zhang F., Huang X., Ma L., Feng L., Zhang R., Wang Y., Ragg H., Zheng Y., and Zhou A. (2016) Heparin binds lamprey angiotensinogen and promotes thrombin inhibition through a template mechanism. J. Biol. Chem. 291, 24900–24911 10.1074/jbc.M116.725895 27681598 PMC5122762

[33] Mangan M. S. J., Bird C. H., Kaiserman D., Matthews A. Y., Hitchen C., Steer D. L., Thompson P. E., and Bird P. I. (2016) A novel serpin regulatory mechanism: SerpinB9 is reversibly inhibited by vicinal disulfide bond formation in the reactive center loop. J. Biol. Chem. 291, 3626–3638 10.1074/jbc.M115.699298 26670609 PMC4751400

[34] Rahgozar S., Amirian T., Qi M., Shahshahan Z., Entezar-E-Ghaem M., Ghasemi Tehrani H., Miroliaei M., Krilis S. A., and Giannakopoulos B. (2015) Improved assay for quantifying a redox form of angiotensinogen as a biomarker for pre-eclampsia : a case-control study. PLoS One 10, e0135905 10.1371/journal.pone.0135905 26312482 PMC4552422

[35] Pol-Fachin L., Franco Becker C., Almeida Guimarães J., and Verli H. (2011) Effects of glycosylation on heparin binding and antithrombin activation by heparin. Proteins 79, 2735–2745 10.1002/prot.23102 21769943

[36] Sumer-Bayraktar Z., Grant O. C., Venkatakrishnan V., Woods R. J., Packer N. H., and Thaysen-Andersen M. (2016) Asn347 glycosylation of corticosteroid-binding globulin fine-tunes the host immune response by modulating proteolysis by *Pseudomonas aeruginosa* and neutrophil elastase. J. Biol. Chem. 291, 17727–17742 10.1074/jbc.M116.735258 27339896 PMC5016167

[37] Rudd P. M., Woods R. J., Wormald M. R., Opdenakker G., Downing A. K., Campbell I. D., and Dwek R. A. (1995) The effects of variable glycosylation on the functional activities of ribonuclease, plasminogen and tissue plasminogen activator. Biochim. Biophys. Acta 1248, 1–10 10.1016/0167-4838(94)00230-E 7711052

[38] Rong P., Campbell D. J., and Skinner S. L. (2003) Hypertension in the (mRen-2)27 rat is not explained by enhanced kinetics of transgenic Ren-2 renin. Hypertension 42, 523–527 10.1161/01.HYP.0000093383.18302.A7 14517223

[39] Lek M., Karczewski K. J., Minikel E. V., Samocha K. E., Banks E., Fennell T., O'Donnell-Luria A. H., Ware J. S., Hill A. J., Cummings B. B., Tukiainen T., Birnbaum D. P., Kosmicki J. A., Duncan L. E., Estrada K., et al (2016) Analysis of protein-coding genetic variation in 60,706 humans. Nature 536, 285–291 10.1038/nature19057 27535533 PMC5018207

[40] D'Arcy A., Villard F., and Marsh M. (2007) An automated microseed matrix-screening method for protein crystallization. Acta Crystallogr. D Biol. Crystallogr. 63, 550–554 10.1107/S0907444907007652 17372361

[41] Battye T. G. G., Kontogiannis L., Johnson O., Powell H. R., and Leslie A. G. W. (2011) iMOSFLM: a new graphical interface for diffraction-image processing with MOSFLM. Acta Crystallogr. D Biol. Crystallogr. 67, 271–281 10.1107/S0907444910048675 21460445 PMC3069742

[42] Evans P. R. (2011) An introduction to data reduction: space-group determination, scaling and intensity statistics. Acta Crystallogr. D Biol. Crystallogr. 67, 282–292 10.1107/S090744491003982X 21460446 PMC3069743

[43] Evans P. R., and Murshudov G. N. (2013) How good are my data and what is the resolution? Acta Crystallogr. D Biol. Crystallogr. 69, 1204–1214 10.1107/S0907444913000061 23793146 PMC3689523

[44] Karplus P. A., and Diederichs K. (2012) Linking crystallographic model and data quality. Science 336, 1030–1033 10.1126/science.1218231 22628654 PMC3457925

[45] McCoy A. J., Grosse-Kunstleve R. W., Adams P. D., Winn M. D., Storoni L. C., and Read R. J. (2007) Phaser crystallographic software. J. Appl. Crystallogr. 40, 658–674 10.1107/S0021889807021206 19461840 PMC2483472

[46] Emsley P., Lohkamp B., Scott W. G., and Cowtan K. (2010) Features and development of Coot. Acta Crystallogr. D Biol. Crystallogr. 66, 486–501 10.1107/S0907444910007493 20383002 PMC2852313

[47] Adams P. D., Afonine P. V., Bunkóczi G., Chen V. B., Davis I. W., Echols N., Headd J. J., Hung L.-W., Kapral G. J., Grosse-Kunstleve R. W., McCoy A. J., Moriarty N. W., Oeffner R., Read R. J., Richardson D. C., et al (2010) PHENIX: a comprehensive Python-based system for macromolecular structure solution. Acta Crystallogr. D Biol. Crystallogr. 66, 213–221 10.1107/S0907444909052925 20124702 PMC2815670

[48] Winn M. D., Isupov M. N., and Murshudov G. N. (2001) Use of TLS parameters to model anisotropic displacements in macromolecular refinement. Acta Crystallogr. D Biol. Crystallogr. 57, 122–133 10.1107/S0907444900014736 11134934

[49] Chen V. B., Arendall W. B. 3rd, Headd J. J., Keedy D. A., Immormino R. M., Kapral G. J., Murray L. W., Richardson J. S., and Richardson D. C. (2010) MolProbity: all-atom structure validation for macromolecular crystallography. Acta Crystallogr. D Biol. Crystallogr. 66, 12–21 10.1107/S0907444909042073 20057044 PMC2803126

[50] Lütteke T. (2009) Analysis and validation of carbohydrate three-dimensional structures. Acta Crystallogr. D Biol. Crystallogr. 65, 156–168 10.1107/S0907444909001905 19171971 PMC2631634

[51] Lütteke T., Frank M., and von der Lieth C.-W. (2005) Carbohydrate structure suite (CSS): analysis of carbohydrate 3D structures derived from the PDB. Nucleic Acids Res. 33, D242–D246 15608187 10.1093/nar/gki013 PMC539967

[52] DeLano W. L. (2010) The PyMOL Molecular Graphics System, version 1.3, Schrödinger, LLC, New York

[53] Krissinel E., and Henrick K. (2007) Inference of macromolecular assemblies from crystalline state. J. Mol. Biol. 372, 774–797 10.1016/j.jmb.2007.05.022 17681537

[54] Sélo I., Négroni L., Créminon C., Grassi J., and Wal J. M. (1996) Preferential labeling of α-amino N-terminal groups in peptides by biotin: application to the detection of specific anti-peptide antibodies by enzyme immunoassays. J. Immunol. Methods 199, 127–138 10.1016/S0022-1759(96)00173-1 8982354

