# Supplementary material for: Structural basis for the specificity of renin-mediated angiotensinogen cleavage
Source: J Biol Chem. 2018 Dec 18;294(7):2353–64. doi: 10.1074/jbc.RA118.006608 (PMC6378967; doi:10.1074/jbc.RA118.006608)
Supplement: Supporting Information [file supp_294_7_2353__index.html]

Structural basis for the specificity of renin-mediated angiotensinogen cleavage — Specificity of angiotensinogen cleavage by renin — Structural basis for the specificity of renin-mediated angiotensinogen cleavage — Specificity of angiotensinogen cleavage by renin — Supporting Information 

# Structural basis for the specificity of renin-mediated angiotensinogen cleavage

## Supporting Information

- Supporting Information (to be published online) - Supporting information table and figures
