# Supplementary material for: Structural basis for the specificity of renin-mediated angiotensinogen cleavage
Source: J Biol Chem. 2018 Dec 18;294(7):2353–64. doi: 10.1074/jbc.RA118.006608 (PMC6378967; doi:10.1074/jbc.RA118.006608)
Supplement: Supporting Information [file supp_RA118.006608_141806_1_supp_251805_pjq7l0.docx]

# Supporting information

# Structural basis for the specificity of angiotensinogen cleavage by renin

**Yahui Yan^1^ (严亚慧), Aiwu Zhou^2*^(周爱武), Robin W. Carrell^1^, Randy J. Read^1*^**

## Table S-1. Primer sequences

| **Name** | **5’-3’ sequence** |
| --- | --- |
| Human AGT-C18S-forward | CACAATGAGAGTACCTcTGAGCAGCTGGCAAAG |
| Human AGT-C18S-reverse | CTTTGCCAGCTGCTCAGAGGTACTCTCATTGTG |
| Human AGT-C138S-forward | TGGAAGGACAAGAACTcCACCTCCCGGCTGGAT |
| Human AGT-C138S-reverse | ATCCAGCCGGGAGGTGGAGTTCTTGTCCTTCCA |
| Human AGT-C232S-forward | TGGAAGACTGGCTcCTCCCTGATGGGAGCC |
| Human AGT-C232S-reverse | GGCTCCCATCAGGGAGgAGCCAGTCTTCCA |
| Human AGT-C308S-forward | TTCACTGAGAGCGCCTcCCTGCTGCTGATCCA |
| Human AGT-C308S-reverse | TGGATCAGCAGCAGGgAGGCGCTCTCAGTGAA |
| Human AGT-N14Q-forward | CACCTCGTCATCCACcAaGAGAGTACCTGTGAG |
| Human AGT-N14Q-reverse | CTCACAGGTACTCTCtTgGTGGATGACGAGGTG |
| Human AGT-N137Q-forward | CCTTGGAAGGACAAGcAgTGCACCTCCCGGCTG |
| Human AGT-N137Q-reverse | CAGCCGGGAGGTGCAcTgCTTGTCCTTCCAAGG |
| Human AGT-N271Q-forward | GAGTTCTGGGTGGACcAgAGCACCTCAGTGTCT |
| Human AGT-N271Q-reverse | AGACACTGAGGTGCTcTgGTCCACCCAGAACTC |
| Human AGT-N295Q-forward | AGTGACATCCAGGACcAgTTCTCGGTGACTCAA |
| Human AGT-N295Q-reverse | TTGAGTCACCGAGAAcTgGTCCTGGATGTCACT |
| Human AGT-R83H-forward | AACTTCTTGGGCTTCCaTATATATGGCATGCAC |
| Human AGT-R83H-reverse | GTGCATGCCATATATAtGGAAGCCCAAGAAGTT |
| Human AGT-N331D-forward | CTCACTTTCCAGCAAgACTCCCTCAACTGGATG |
| Human AGT-N331D-reverse | CATCCAGTTGAGGGAGTcTTGCTGGAAAGTGAG |
| Human AGT-N334T-forward | CAGCAAAACTCCCTCAcCTGGATGAAGAAACTA |
| Human AGT-N334T-reverse | TAGTTTCTTCATCCAGgTGAGGGAGTTTTGCTG |
| Human prorenin D226A-forward | TGCCTGGCATTGGTAGcCACCGGTGCATCCTAC |
| Human prorenin D226A-reverse | GTAGGATGCACCGGTGgCTACCAATGCCAGGCA |


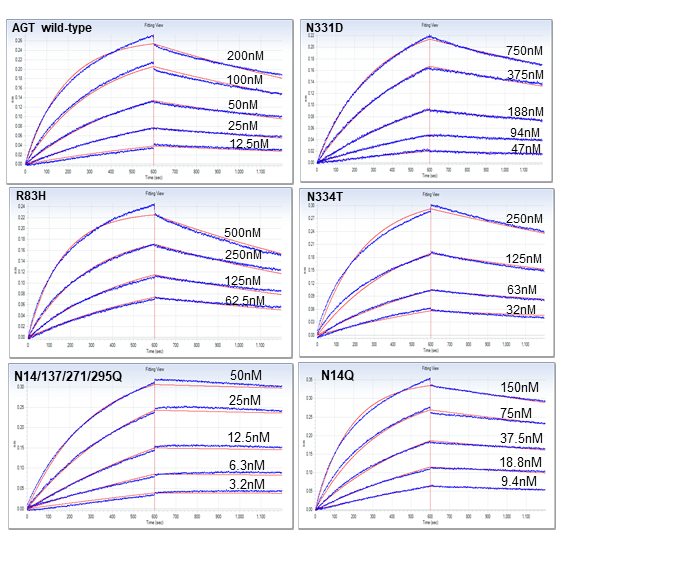


**Figure S-1. Binding affinity measurements of renin and angiotensinogen.** (A) The binding kinetics were measured on a FortéBio Octet Red96 system as described in Methods. The response wavelength shift (nm) was recorded as a function of time (seconds), shown as blue curves. A global fit (red curves) was adopted to calculate *k*_on_, *k*_off_ and *K*_D_ for AGT mutants binding to renin. One representative experiment out of three is shown.


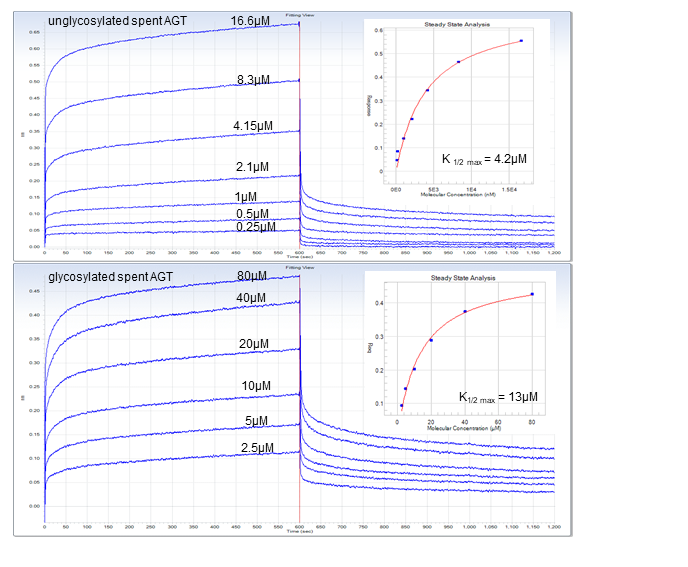


**Figure S-2. The binding kinetics for spent AGT and renin D226A.** The binding kinetics for unglycosylated (A) and glycosylated (B) spent AGT on renin D226A were measured on a FortéBio Octet Red96 system as described in Methods. The response was recorded as a function of time (seconds), shown as blue curves. A steady state analysis shown as inset was used to calculate the *K*_1/2 max_ value due to fast-on and fast-off binding interactions. One representative experiment out of three is shown.


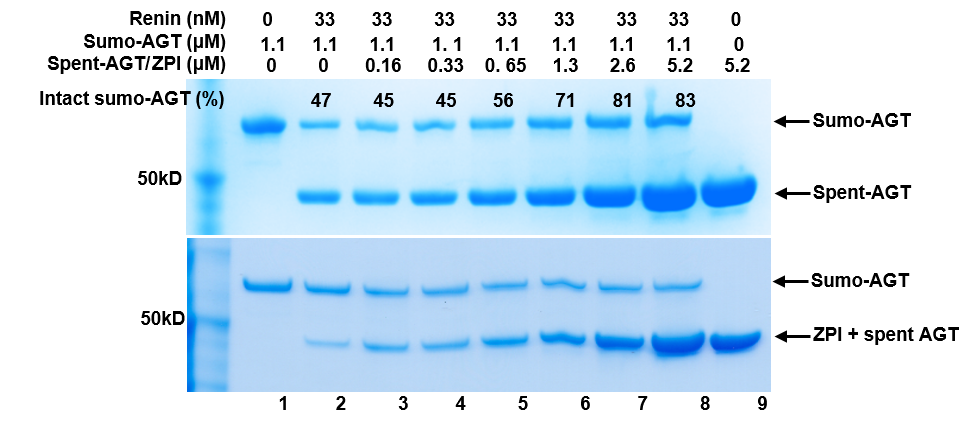


**Figure S-3. The product inhibition effect of spent AGT on renin activity.** (A) A series of 2-fold dilutions (0-5.2μM) of unglycosylated spent-AGT were incubated with 33nM renin and 1.1µM unglycosylated sumo3-AGT at room temperature for 2 hours to test for its inhibition of the renin activity. The percentage of remaining sumo3-AGT was calculated by densitometry. Inhibition became evident when the concentration of spent AGT was 0.65µM (lane 5). Note that *K*_D_ is 4.2 µM for the interaction of unglycosylated AGT and renin D226A (Figure S-2). (B) As a negative control, another serpin, protein Z dependent inhibitor (ZPI), did not show inhibition of cleavage of sumo3-AGT by renin.


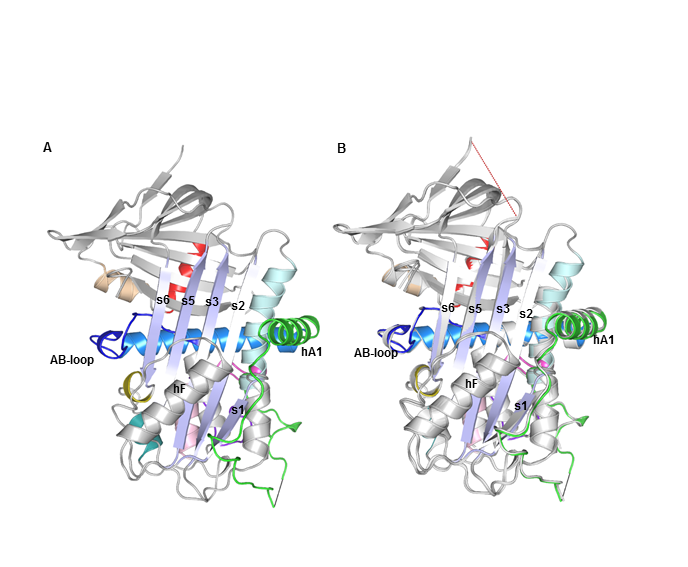


**Figure S-4. The structure of RCL-cleaved AGT has no major conformational change.** (A) Cartoon view of RCL cleaved human AGT. The serpin framework is shown as grey with helix A in marine, the central β sheet with 5 strands (s1, s2, s3, s5, s6) in light blue and the AB-loop connecting helices A and B in blue. The 63-residue N-terminal extension to the serpin framework in AGT is colored as green. (B) The RCL-cleaved AGT superposes on native AGT with an rmsd of 0.28 Å over 371 Cα atoms. The native AGT is shown as grey and the color scheme of the cleaved AGT is the same as in (A). The disordered RCL of native AGT is indicated with red dashes. The central β-sheet of RCL cleaved AGT is tightly folded and embraced by the AB-loop and helix A1 (hA1). There is no major conformational change after the RCL cleavage.


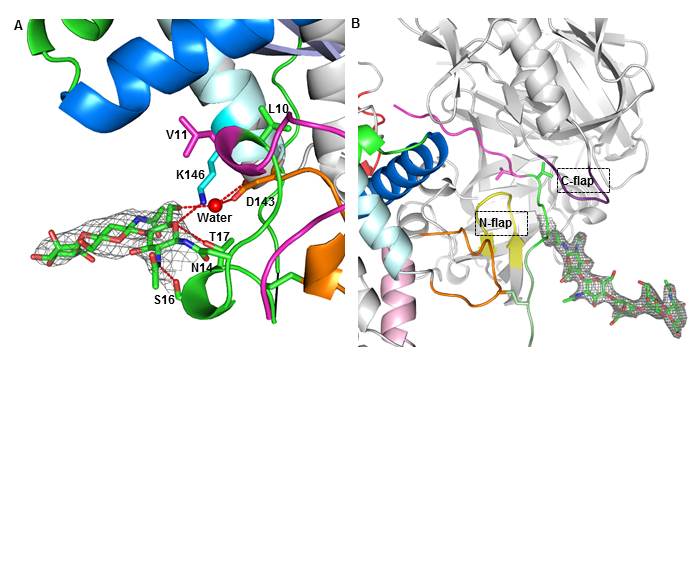


**Figure S-5. Electron densities for glycans in native AGT and AGT in the complex.** (A) In native glycosylated AGT, the oxygen on the first NAG carbohydrate ring forms a hydrogen bond with the nitrogen of K146 of helix D of AGT, and the hydroxyl group forms hydrogen bonds with a water which is stabilized by forming two hydrogen bonds with D143 of AGT. Clear electron density (grey mesh, 2mFo-DFc map, contoured at 1.0 sigma, including density within 1.6 Å of atoms in the glycan) shows the glycan linkage as β-D-Manp-(1-4)-β-D-GlcpNAc-(1-4)-β-D-GlcpNAc-(1-4)-N14. (B) The inserted N-terminal peptide of AGT, covered by the N-flap and C-flap, emerges from the renin active cleft. The glycan attached to N14 of AGT in the complex structure has no interactions with the surrounding renin residues. The glycan linkage is β-D-GlcpNAc-(1-2)-α-D-Manp-(1-3)-β-D-Manp-(1-4)-β-D-GlcpNAc-(1-4)-β-D-GlcpNAc-(1-4)-N14. Electron density contours are shown as for part (A).


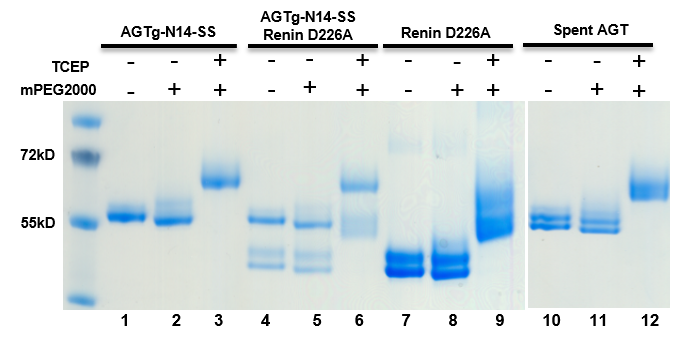


**Figure S-6. Confirmation of the conserved Cys18-Cys138 disulfide bond.** The native AGT, AGT/renin D226A complex and spent AGT were incubated with mPEG2000 in the absence or presence of TCEP. AGT was not modified with mPEG2000 in the absence of TCEP (lanes 2, 5 and 11). After TCEP reduction, the modified AGT migrated more slowly in the SDS-PAGE (lanes 3, 6 and 12), corresponding to two conjugated mPEG-2000 molecules. Similarly, renin D226A was modified by mPEG-2000 after TCEP reduction (lane 9). Note that the two free Cys232 and 308 in AGT were replaced with serine. Therefore, the native AGT, AGT in the complex and spent AGT had an intact disulfide linking Cys18 and Cys138.


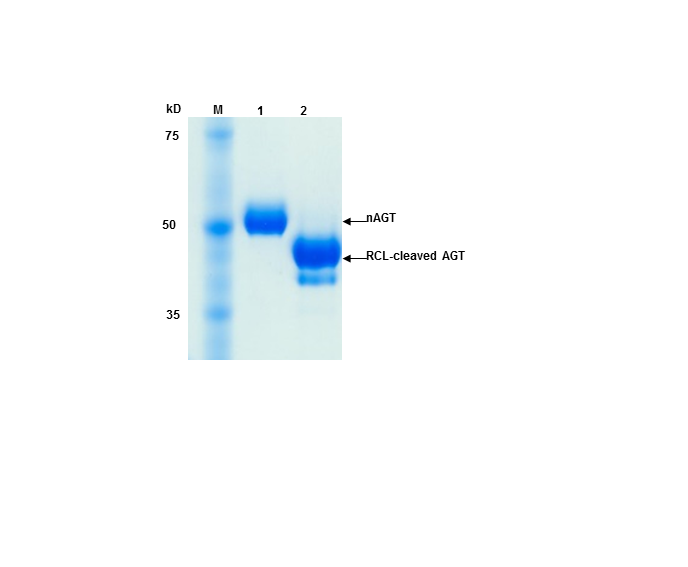


**Figure S-7. The RCL-cleaved human AGT on SDS-PAGE.** AGT cleaved in the RCL (lane 2) by thermolysin migrated faster than intact AGT (lane 1) in SDS-PAGE.
